# Retrospective Epidemiological Study of Achilles Tendon Ruptures

**DOI:** 10.7759/cureus.96019

**Published:** 2025-11-03

**Authors:** Edmond F Iradukunda, Abdul Hadi Kafagi, Mustafa Farooqi, Anand Pillai

**Affiliations:** 1 Faculty of Biology, Medicine and Health, University of Manchester, Manchester, GBR; 2 Trauma and Orthopaedics, Wythenshawe Hospital, Manchester University NHS Foundation Trust, Manchester, GBR

**Keywords:** achilles tendon rupture, epidemiology, prevention, retrospective audit, trauma and sports injury

## Abstract

Whilst the epidemiology of Achilles tendon ruptures (ATRs) is well known in the context of other countries, not many epidemiological studies have been conducted in the UK, demonstrating the lack of recent, reliable data relevant to our population context.

The approach taken was retrospective data collection of ATRs and relevant history from participants’ electronic patient records (EPR), followed by a telephone interview to confirm and investigate data points not included in patient notes.

From a sample of 88 participants, 76% were male, and the median age was 44. The most prevalent activity and mechanism of injury was playing football (39%), and accelerating from a stationary position (30%). ATRs were predominantly sports-related (67%), and the incidence of sports-related ATRs and total ATRs was greatest during summer (34%), followed by spring (30%). The median (IQR) age of participants with sports-related ATRs was 40.0 (35.0 - 45.5) years, compared to 65.5 (54.0 - 73.5) years for non-sports-related ATRs.

In this sample, ATRs most commonly occur in middle-aged men in a sporting context during the spring and summer seasons. Non-sports-related ATR incidence is greater in older age groups. These results are consistent with both local British studies and international studies, but limitations to this study highlight the need for further research with a similar methodology and greater scale, which would aid in identifying high-risk groups that could benefit from tailored injury prevention strategies.

## Introduction

The Achilles tendon is one of the strongest tendons in the human body [1,2]. It is responsible for plantarflexion, as it tethers the gastrocnemius muscle to the calcaneus [1,2]. Due to its vital role in facilitating activities of daily living, Achilles tendon ruptures (ATRs) can be debilitating, and patients can take years to return to full functionality [3]. Not all patients make a complete recovery, with some suffering lasting complications, such as a limp [4].

Most ATRs can be categorised as sports-related or not, with sports-related ATRs (SRATRs) being associated with a single event of impact or trauma leading to a rupture [5-9]. Non-sports-related ATRs (NSRATRs) are often multifactorial, associated with comorbidities and other risk factors [10,11]. SRATRs are associated with middle-aged, physically active men during spring and summer seasons [5,6], whilst NSRATRs are typically associated with comorbid patients [10,12].

At the time of writing, no national British studies focus on the epidemiology of ATRs, although other studies have been done on a similar scale to this study [6]. Therefore, there are no national standards to compare the data collected in this study. Smaller studies, such as the one previously cited, are inappropriate to use as a benchmark standard, as factors such as geographical location, age distribution, and overall patient demographics could significantly impact the results found in this study and others of similar aims.

This study aims to describe the epidemiology of ATRs within the trust. The data collected in this project may inform future audits on treatment outcomes and aid in developing hypotheses on the epidemiology and prognosis of patients with ATRs presenting to the trust of this study. Epidemiological research gives an insight into the demographics of ATR in the UK, which could guide national treatment pathways and identify modifiable risk factors that could be targeted by population health strategies.

It could be argued that this data should initially be used to compare the outcomes of conservative and surgical treatment of ATR. However, strong evidence supports the outcomes of both conservative and surgical treatment of ATR being similar [13]. Therefore, the evidence presented in this retrospective audit would be insufficient to disprove the evidence supporting this hypothesis.

The overwhelming majority of the reviewed literature conveyed the following findings: most participants with ATRs are male, most ATRs are sports-related, and acceleration from a stationary position was amongst the most common of mechanisms of injury [8,14]. The median and mean ages of incidence were found to be between 41 and 50, respectively [5,6,9,15,16]. Comorbidities were found to be more common in patients with NSRATRs. The majority of participants in Houshian et al. [9] were managed surgically, although in Briggs-Price et al. [6], which is a British study where treatment pathways are most likely to be similar to this study, a small minority of participants were managed surgically, with conservative management being first line in most cases. Seasonal variation was found in some studies, supporting increased incidence during the spring and summer seasons [5].

By adapting the methods of other epidemiological studies, this study hopes to describe the epidemiology of ATRs within the trust.

To summarise, this project is a single-site epidemiological overview of ATRs. The data retrospectively collected will be analysed and presented, followed by a discussion of factors causing the distribution of incidence.

## Materials and methods

This study, conducted at Manchester University NHS Foundation Trust, started by retrospectively collecting data from electronic patient records (EPR), followed by a brief telephone consultation to clarify any ambiguous or missing information.

EPR

The sample contained patients with ankle injuries from July 2023 to April 2025, spanning 21 months. Inclusion criteria included all patients diagnosed with ATRs within the previously mentioned timeframe. Exclusion criteria included: patients with ATRs outside the timeframe, patients with gastrocnemius tears and patients misdiagnosed with ATRs. Misdiagnosis was identified through patient notes, where it was explicitly noted that the patient was misdiagnosed. Exclusions from the given sample reduced the sample size to 88 patients. The EPR used was HIVE, where quantitative and qualitative data were collected manually. Qualitative data were collected by reading through all the admission notes and clinic letters related to the ATR. For example, treatment was determined by the presence or absence of surgical operation notes. 

Telephone consultation

Data that could not be ascertained from patient records was collected via telephone consultation. Patients were questioned using a set proforma to clarify and confirm the most critical variables in the study (Appendix). This served to identify any errors in documentation in the EPR or errors in the first stage of data collection from the EPR. Patients who reported factors that were exclusion criteria were excluded, and when data between stage one and stage two of data collection conflicted, the data from the telephone consultation was prioritised. Three attempts were made to contact patients, one per day, until telephone consultation attempts were discontinued. 

Mechanism of injury classification

Mechanisms of Injury were strictly classified, based on classifications from similar studies that used video analysis to determine the mechanism of injury [14,17,18]. It was decided to follow these classifications developed by other studies, as their methods of analysis were more reliable and objective than the methods used in this study. For example, Yüce et al. [17] were able to define a mechanism of injury based on the velocity of the patient and the positioning of the ankle joints using video footage that was scrutinised by three analysts. Mechanisms of injury were defined as: acceleration from a stationary position, vertical jumping, landing, twisting, fall trauma, deceleration, running, other plantarflexion, and other forced dorsiflexion. These categories were developed before data collection and further refined after telephone consultations to accommodate all the mechanisms of injuries that were encountered. For example, plantarflexion and dorsiflexion that did not adequately satisfy the criterion of the first seven classifications were consequently classified as other plantarflexion and other forced dorsiflexion, respectively. 

This study did not have any video footage for analysis, but mechanisms of injury were determined based on the data collected in the first and second phases of data collection. Using the patient's recount of events, the telephone interviewer would clarify ankle positioning and velocity to determine the mechanism of injury. Where these details were inconclusive and did not fit within the mechanisms of injury, the mechanism of injury was determined to be unknown and excluded from data analysis.

Analysis

The results were analysed within Microsoft Excel, using built-in functions to calculate medians, means, standard deviations (SD), and interquartile ranges (IQR).

## Results

Sample demographics

The final sample size was 88 patients after exclusions, of which 76% (n=67, 95% CI 67.2-85.0%) were male. The sample's median age (IQR) was 44 (37 - 61.3) years. The median (IQR) wait time before presenting to A&E was one (zero - three) day(s), based on data collected from 87 out of the 88 participants. The mean (standard deviation) BMI of the participants was 28.50 (5.88) for male participants and 29.1 (6.14) for female participants (Table 1).

**Table 1:** Sample Demographics This table shows the demographics of the sample used after unsuitable candidates were excluded

Sex	Participants n (%)	Median Age (IQR), years	Mean BMI ± SD, kg/m^2^
Male	67 (76)	42.0 (36.0 - 53.5)	28.5 ± 5.88
Female	21 (24)	53.0 (44.0 - 66.0)	29.1 ± 6.14
Total	88 (100)	44.0 (37.0 - 61.3)	28.6 ± 5.89

Contacting and consenting

**Figure 1:**
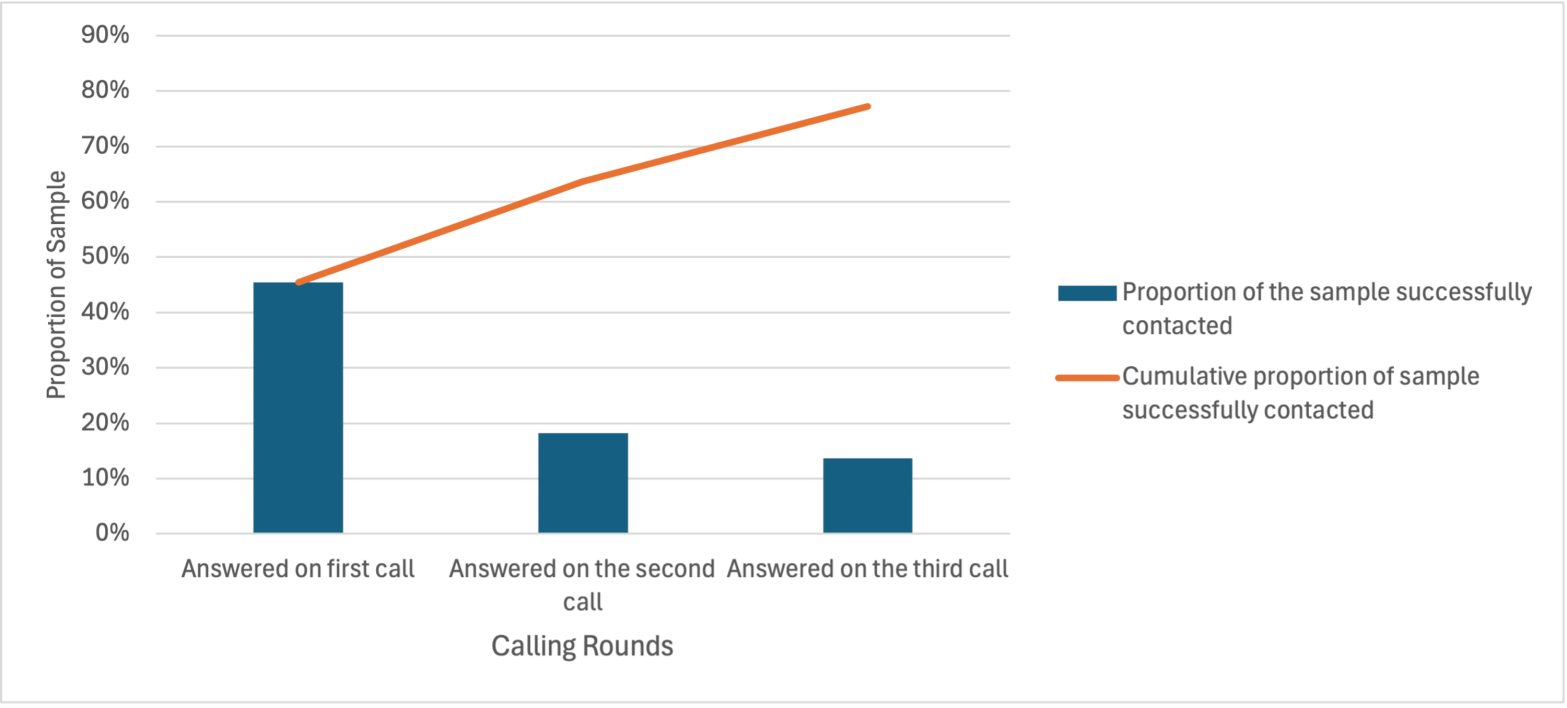
Calling Attempts and Success Rates A graph showing the proportion and cumulative proportion of the sample successfully contacted in each round of calling

**Figure 2:**
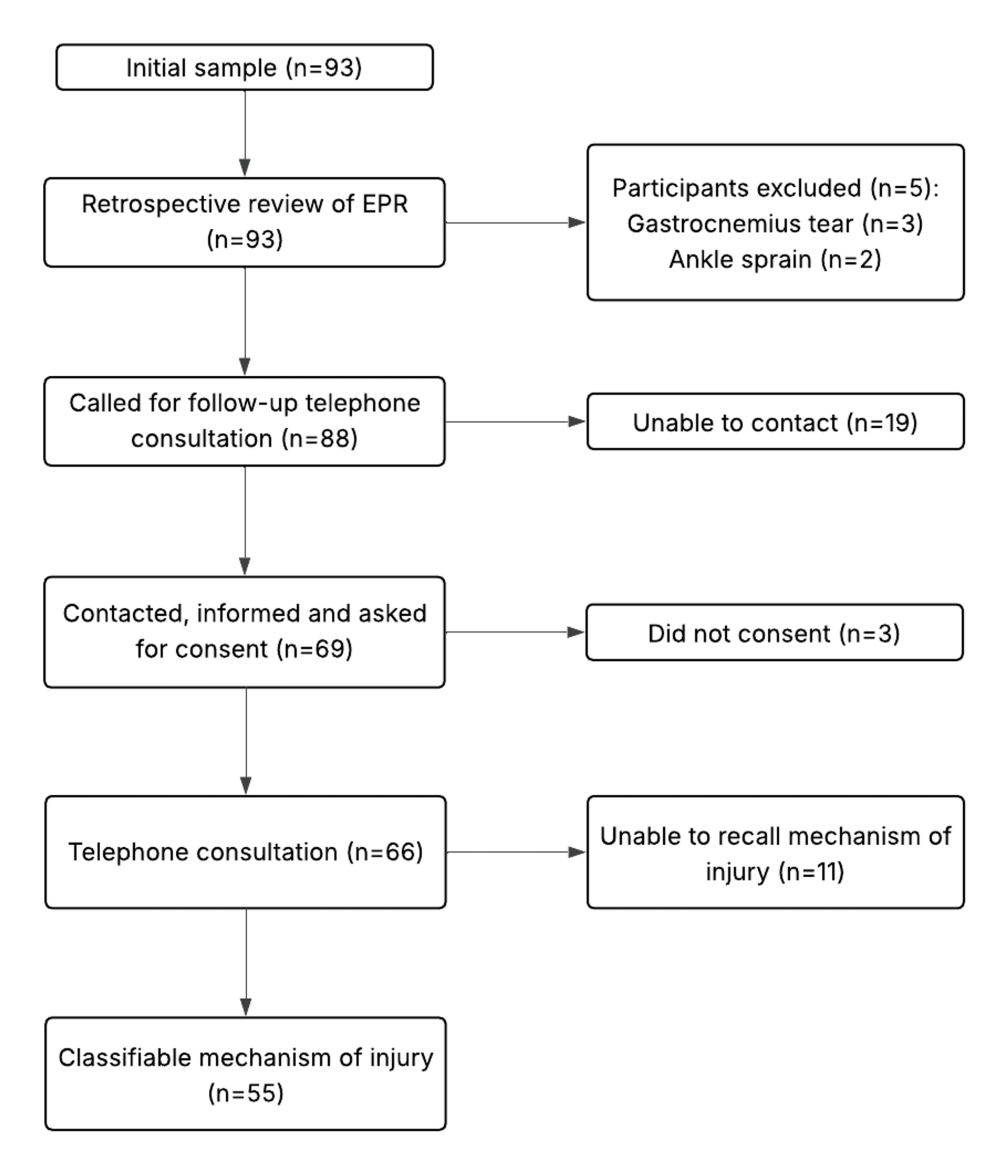
Participant Flowchart A flowchart illustrating the number of participants included in each part of the study

Around 78% of participants (n=69/88) were successfully contacted in the second stage of data collection. This was achieved by calling patients in three rounds across five days. In the first round, 45% (n=40/88) of consultations were conducted. In the second round, 18% (n=16/88) of participants were successfully contacted, and in the third round, 14% (n=12/88) of participants were spoken to (Figure 1). One of the participants was an inpatient and spoken to in person, bringing the total to 69 contacted participants, of which 96% (n = 66/69) consented to discuss their ATR in detail (Figure 2).

Gender proportions between the contacted and non-contacted groups were similar (Table 2), and although the median ages appeared to vary between them, with the median (IQR) age of the contacted group being 47 (38.0 - 62.0) and the non-contacted group 37 (34 - 46.3), this result was not statistically significant, U = 490, Z = 1.68, p = 0.09 (two-tailed). Similarly, the mean BMI difference was insignificant, U=278.5, z= 1.00, p=0.32 (two-tailed). Overall, demographics were similar between contacted and non-contacted groups.

**Table 2:** Demographics of Contacted and Not Contacted Participants This table compares the demographics of contacted and non-contacted participants to the overall sample

Groups	Frequency n (%)	Male Participants n (%)	Female Participants n (%)	Median Age (IQR)	Mean BMI ± SD	Surgical Treatment n (%)
Contacted	69 (78%)	52 (75%)	17 (25%)	47.0 (38.0 - 62.0)	29.0 ± 5.99	8 (12%)
Non-contacted	19 (22%)	15 (79%)	4 (21%)	37.0 (34.0 - 46.3)	25.2 ± 9.22	1 (5%)
Total	88 (100%)	67 (76%)	21 (24%)	44.0 (37.0 - 61.3)	28.6 ± 5.89	9 (10%)

Mechanism of injury data was collected from 83% (n = 55/66) of the consenting participants, with the remainder uncertain of the mechanism of their injury (Figure 2). Of the 22% (n=19/88) of participants that were not successfully contacted, 79% (n=15/19) had undetermined mechanisms of injury.

SRATRs vs NSRATRs

The most prevalent activities of injury were sports-related at 67% (n = 59, 95% CI 56.2-76.9%), of which the most common sport was football (Table 3). Of the non-sport-related activities of injury, the most common activity was walking. The median age (IQR) of patients presenting with SRATRs was 40.0 (35.0 - 45.5) compared to those with NSRATRs at 65.5 (54.0 - 73.5) years old, which was found to be statistically different (U = 232, p < 0.05). ATRs most commonly affected the left Achilles tendons in SRATRs (n = 36/59, 61%) and NSRATRs (n = 16/29, 55%).

**Table 3:** Activity of Injury Proportions A table showing the proportion of activities of injury relative to the sample

Activity of Injury	N	%
Football	34	39%
Other sports	18	20%
Walking	10	11%
Descending stairs	4	5%
Netball	4	5%
Tennis	3	3%
Fall	3	3%
Tripped	2	2%
Manual labour	2	2%
Standing	2	2%
Ascending stairs	2	2%
Not known	1	1%
E-scooter	1	1%
Jumping	1	1%
Sitting down	1	1%
Grand total	88	100%

The dataset's most common months for ATRs were summer at 34% (n = 30, 95% CI 24.2-44.0%) and spring at 30% (n = 26, 95% CI 20.0-39.1%). SRATRs were more prevalent in spring and summer than in autumn and winter. There is no significant seasonal variation between SRATRs and NSRATRs based on this dataset (x^2^(3) = 3.28, p = 0.351). 

**Figure 3:**
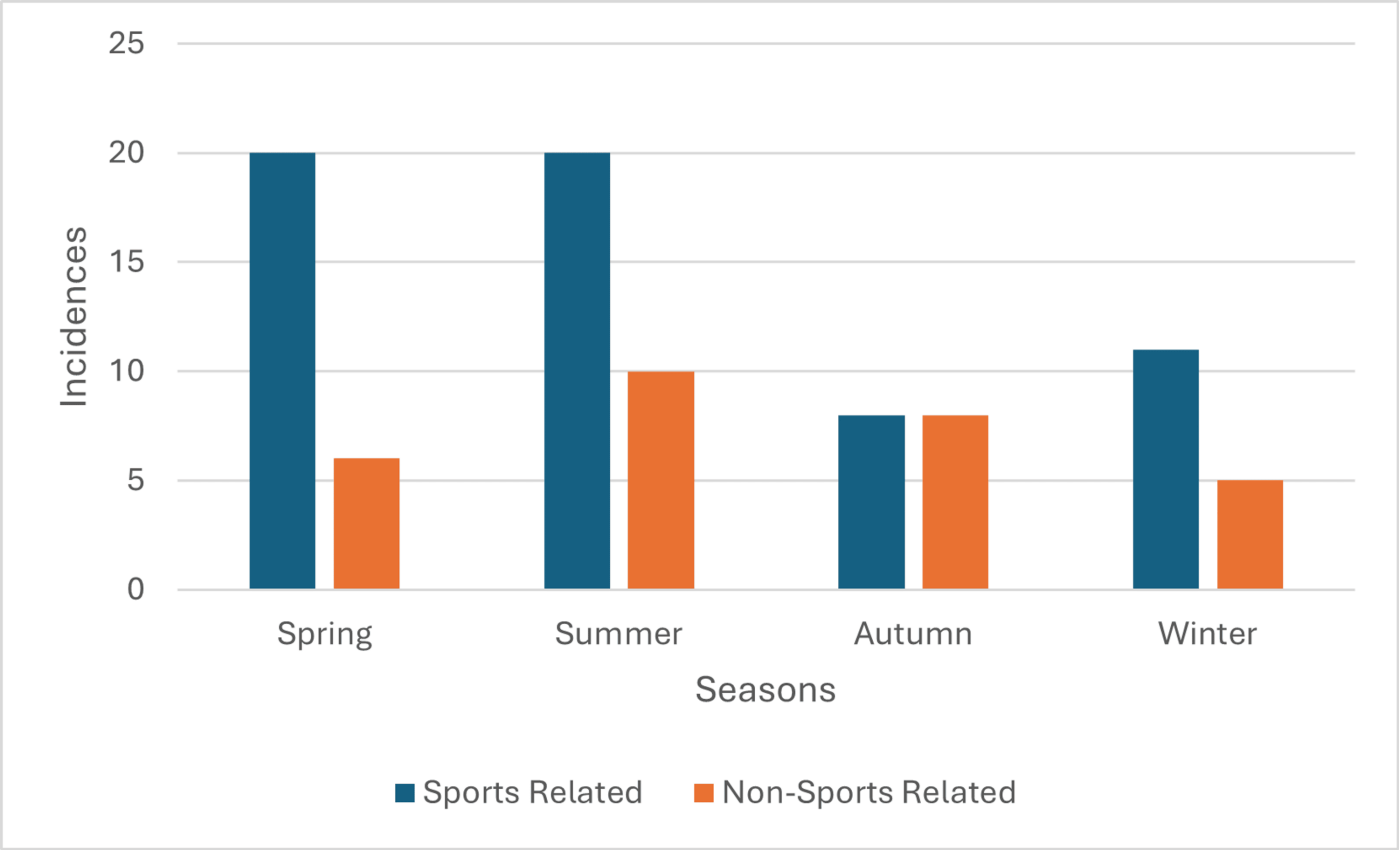
Seasonal Variation of Achilles Tendon Rupture Incidence A graph illustrating the incidence of SRATRs and NSRATRs across different seasons.

Mechanisms of injury

**Figure 4:**
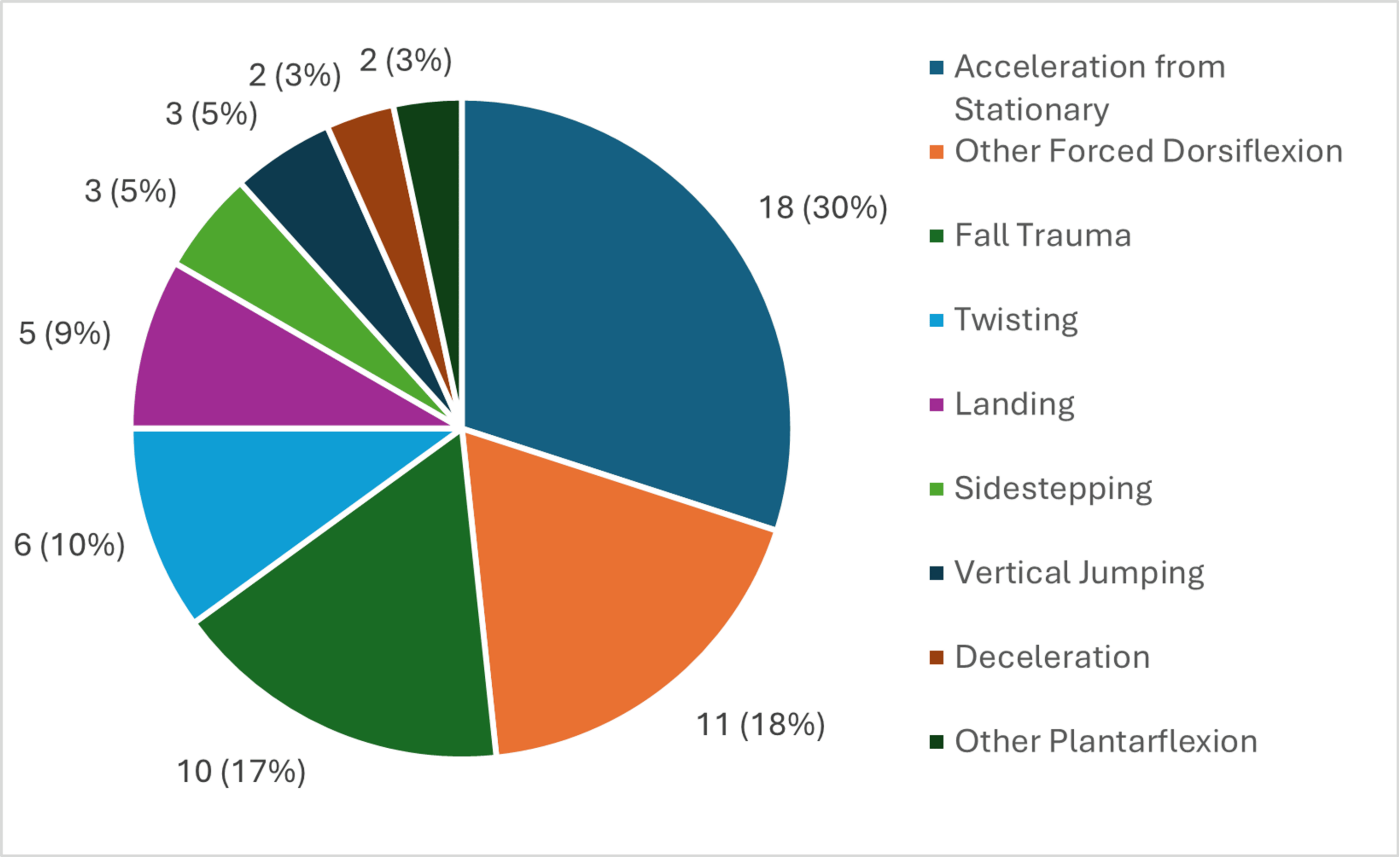
Proportions of Mechanisms of Injury of Achilles Tendon Ruptures A pie chart illustrating the frequency of mechanisms of injury, n (%), relative to the total known mechanisms of injury.

The most common mechanism of injury was acceleration from a stationary position, followed by other forced dorsiflexion mechanisms of injury (Figure 4). For NSRATRs, the most common mechanism of injury was fall trauma (Figure 4).

Past medical history

Previous Pain and Ruptures

Around 24% (n=21/88) of participants reported ipsilateral ankle pain preceding their ATRs, most commonly caused by strain from previous physical exertion. The median (IQR) length of pain preceding the ATR was 56.0 (12.5 - 217.5) days. Approximately 10% (n=9/88) of participants reported previous ATRs, of which the majority (n=8/9) were contralateral.

Comorbidities and Risk Factors

**Figure 5:**
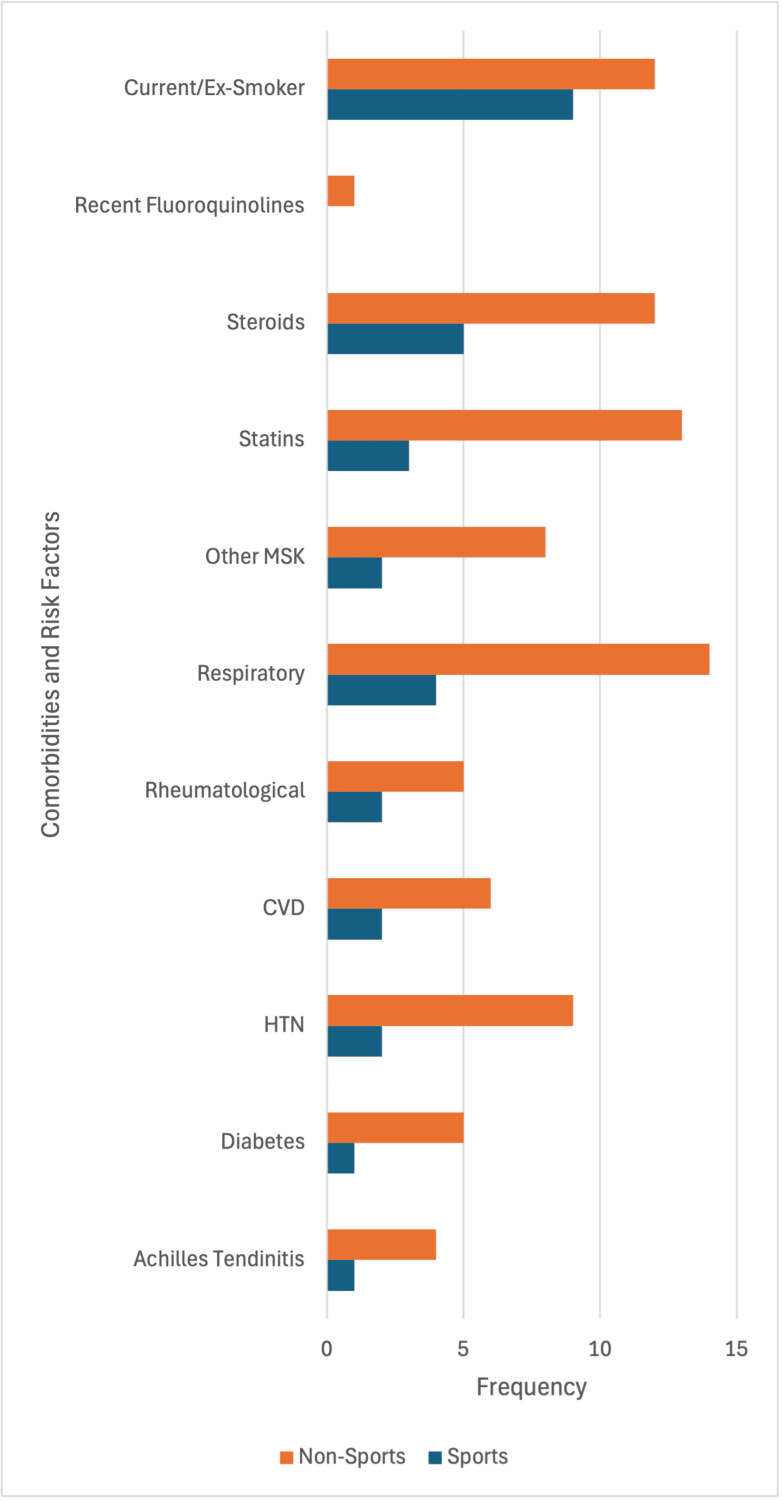
Risk Factor Prevalence A graph demonstrating the incidence of ATR risk factors in SRATRs and NSRATRs

Around 24% (n=21/88) of participants reported being current or ex-smokers. All recorded comorbidities and risk factors were most prevalent in participants with NSRATRs, including Achilles tendinitis, with four out of five incidences occurring in NSRATRs (Figure 5). By mean (SD), patients had 0.7 (1.1) comorbidities, with participants with NSRATRs having a mean of 1.6 (1.1) comorbidities and participants with SRATRs having a mean of 0.2 (0.6) comorbidities. Mean BMIs (SD) between the two ATRs were 26.6 (4.28) for SRATRs and 31.3 (6.70) for NSRATRs.

Treatment and re-rupture

Only 10% of participants (n=9/88) were surgically managed, with the majority managed conservatively. Of all participants, only 5% (n=4/88) proceeded to re-rupture their Achilles tendon.

## Discussion

Summary of findings

The results showed that ATRs within the sample are most prevalent in middle-aged men playing football during spring and summer, accelerating from a stationary position. This is consistent with the reviewed literature.

Demographic Findings

The median (IQR) age of the sample used was 44.0 (37.0 - 61.3), which is within the age ranges and similar to the medians found in similar studies [5,6,9,15,16]. Similar to the cited literature, the majority of the participants in this study were also male, highlighting the increased incidence of ATRs within the male sex, which Houshian et al. [9] suggested could be due to the increased participation rate in sports, depending on the country of the study.

SRATR vs NSRATR Findings

Around 67% (n = 59, 95% CI 56.2-76.9) of ATRs in this study were sports-related. Whilst limitations in this study affect the application and interpretation of the results, the proportion found was similar to the 75% found in Hoshian et al. [9], and 65.2% found by Briggs-Price et al. [6]. A similar population easily explains the similarity between the latter study, as both studies took place within the UK in urban areas. The similarity between the former study could also be explained by the fact that the UK and Denmark share similar demographics, both being highly economically developed European countries.

The median (IQR) ages of participants with SRATRs were similar to those of Briggs-Price et al. [6], at 40.0 (35.0 - 45.5) and 41 (12.2), respectively. However, the median ages of participants with NSRATRs were 65.5 (54.0 - 73.5) and 55.4 (13.4), respectively, which is a significantly greater difference. One explanation for this could be the smaller sample size of this study, in which such a small proportion of NSRATRs may not accurately represent the population. 

In both studies, the results showed that SRATRs were less likely to have preceding Achilles pain and comorbidities [6].

Mechanisms of Injury

In support of Briggs-Price et al. and Park et al. [5,6], the most common mechanism of injury was acceleration from a stationary position in this study, and football was observed to be the most common activity of injury. The predominance of football-related ATRs likely reflects the local population where football is a popular recreational sport. Certain activities, such as football, require players to react quickly by rapidly accelerating from a stationary position, as explained in Villa et al. [14], which may explain this observation.

Risk Factors

This study’s incidence of risk factors was found to be lower than that of Briggs-Price et al. [6]. The cause for this disparity is multifactorial, including changes in methodology, this study having a relatively smaller sample size, and selection bias from both studies. The most significant probable factor is that the comorbidity count calculated in this study only consisted of the conditions included in the questionnaire. It could be assumed that Briggs-Price et al. [6] included all of their participants’ comorbidities.

Preceding Pain

The proportion of participants experiencing ankle pain preceding the rupture significantly differed from Briggs-Price et al. [6] at 24% and 54%, respectively. Given that the comorbidity count was greater in participants with preceding ankle pain than those without at 0.8 to 0.6, respectively, this difference in preceding ankle pain may be potentiated by the increased comorbidity count in Briggs-Price et al. [6]. A positive correlation between comorbidity count and preceding ankle pain incidence could be explained by the multiple risk factors that affect tendon development and maintenance [10], in which such comorbidities could not only cause ATRs but also induce Achilles tendinopathy, presenting as that pain before rupturing.

Limitations

Sample

A sample size of 88 is insufficient to generalise the ATRs presenting to an NHS trust. This sample was taken from patients presenting to a single site for physiotherapy and is therefore exposed to selection and exclusion bias. Patients excluded from this sample may include those engaging in physiotherapy at neighbouring hospitals, who did not undergo physiotherapy, who absconded or self-discharged, and who underwent private physiotherapy. This means milder cases of ATR and ATRs in participants with access to private healthcare, where the epidemiology and mechanisms of injury may have differed from the results found in this study, were not adequately represented, significantly limiting the size and reliability of the data collected from this sample.

Therefore, the total number of ATRs is unknown, and the sample used may not appropriately or proportionally represent all the patients with ATRs presenting to the trust.

EPR

Data was collected manually from EPR, introducing the risk of human error. This was demonstrated when confirming patient histories during telephone consultations with the participants. Exporting data directly from the EPR would have eliminated the risk of human error from collecting quantitative data. Fortunately, the second stage of data collection partially mitigates the risk of human error introduced by manual data collection.

The second stage of data collection also highlighted inaccuracies and errors in participants' records. Participants who were contactable and consented to the consultation were able to identify these errors. A significant portion of the sample was not contactable during the second stage of data collection, which may have introduced non-response bias. Statistical analysis was able to determine that there were no significant differences in the demographics between contacted and non-contacted groups. Regardless, the exclusion or lack of data from the non-contacted group means that there is an irrefutable degree of non-response bias in this study, which is more likely to have particularly affected data regarding the activities and mechanisms of injury, which often required further elaboration during telephone consultations due to the lack of detail in records.

Telephone Consultations

Telephone consultations were conducted during the second phase of data collection. These were carried out using clinic telephones in the unused clinic rooms during working hours. Calling only during working hours affected the availability of most participants, given that the sample's median age is below retirement age. This is demonstrated by the fact that, at the highest level, only 45% of participants were contactable in any round of calls.

In addition, many participants gave feedback that they received a voicemail but could not call back to contact the data collector. This was due to the data collection occurring in different clinic rooms on different phones, with no assigned contact number for participants. Overall, the method used to contact patients was negatively affected by both place and time, limiting the total number of participants contacted. This may have affected the study, with over half of the unknown mechanisms being from uncontacted participants.

Analysis

In this study, the mechanism of injury was ascertained using EPR notes and telephone interviews. This introduced limited-response and recall biases, as some patients needed prompting to trigger their recollection of events. Prompting may have encouraged patients to give a mechanism of injury that fit within the predefined mechanisms of injury inappropriately. In comparison, many studies that specifically focus on the mechanism of injuries use video analysis to determine the mechanism of injury. Video analysis is a data collection method of significantly increased reliability and objectivity compared to telephone interviews and recounts from patient notes. In the context of the NHS and this study, video analysis is impractical, given that not many patients would have video footage of their injuries.

Multivariate regression was not carried out due to the limited sample size and proportion of missing data, which would have reduced its reliability by further reducing the sample size, overvaluing outliers in its analysis, and overfitting. This constrains our interpretations of the results as risk factors cannot be statistically proven to correlate with particular outcomes or ATR incidence in the context of multiple independent variables. Future studies with larger samples following the methodology of this study should aim to employ multivariate regression in order to elicit trends and establish confounding factors of ATR incidence.

Overall, the limitations of this study are due to a small sample size and a method of data collection that is significantly vulnerable to bias. This greatly affects the reliability of the findings in the context of the sample and restricts the interpretations of the results. The results from this sample may not reliably represent the entire population of patients with ATRs presenting to the site. The statistical analysis carried out sufficed for observational purposes, but was vulnerable due to confounding and therefore not adequate for establishing independent risk factors of ATRs or drawing causal inferences.

Recommendations

*Increase Sample Size*

Trust-wide: One of the most significant limiting factors affecting this study's reliability is its sample size. Sample size increases the data analysis's reliability and helps elicit trends between conditions with relatively low incidence, such as Achilles tendinopathy or drug-induced tendinopathy from recent fluoroquinolone use. Similar studies state sample sizes of greater than 300 [8,9,19,20]. A greater sample could be gathered by either expanding the range of the sample to include all ATRs within the trust, increasing the date range further back than 2023, or both of the provided options.

Nationwide: At the time of writing, there are no national publications on the epidemiology of ATRs native to the UK, although there are similar publications in many other countries [5,8,9]. In addition to repeating the study across the entire trust, it would be beneficial to carry out a study with a much larger sample of the nation or country. The results of this study could guide nationwide public health campaigns, such as increasing awareness of the risk of ATR on fluoroquinolones. It would also highlight differences in ATR epidemiology compared to other countries, which could be used as teaching points for newly starting international medical graduates (IMGs) and migrating doctors to aid their clinical judgement. A national study of ATR epidemiology would not have to be undertaken as one sole project - similar studies can be done for trusts around the country, which could then be coalesced into a meta-analysis for the country.

Evaluating the Effect of Terrain and Footwear on Injury Incidence in Football

As football was the activity of injury of greatest incidence, further studies into ATRs playing football have the greatest potential to increase our understanding of ATRs and their incidence. Footwear is known to significantly affect running biomechanics, although it has not been confirmed whether this affects the risk of musculoskeletal injury [21-23]. A study focusing on either of these could help inform professional and casual players on the risks and benefits of different footwear or terrain when playing football.

Improving Documentation

During phase one of data collection, many inaccuracies were found on EPRs, as well as unfilled data fields for patient history, conditions, and smoking history, despite the history being known and detailed in the patient's admission and clinic notes. This highlighted a lack of understanding of the full EPR's functions or poor documentation within the trust. Audits and quality improvement projects (QIPs) could correct and improve this. If the aforementioned data fields were filled regularly, it could not only increase the speed at which data could be manually collected for research but also provide a reliable source of information that is verified by multiple members of multidisciplinary teams for patient histories, rather than having to predominantly rely on the most recent notes of a single healthcare professional.

Patient Education on Recovery Duration

When conducting telephone interviews, many patients expressed dissatisfaction at not feeling adequately informed about the length of recovery from ATRs. Patients often testified to frustration or disappointment at their slow recoveries. They could distinguish between their satisfaction with their quality of care and their disappointment at not feeling thoroughly informed about the length of their recovery. Based on this feedback, it would be most appropriate to commission the design of new patient information on ATRs. This could take the form of patient leaflets, to be used locally at the single site or across the entire trust or nationally, seeing as the NHS patient information website does not have a page on Achilles tendon ruptures at the time of writing, despite having a page for heel pain and listing ATRs as a differential [24].

Targeted Injury Prevention

The most common activity of injury in this dataset was football. If the following studies supported a hypothesis of football being a predominant activity of injury within the trust, an effective public health campaign focusing on injury prevention in areas such as astro-turf football pitches for hire would significantly reduce ATR incidence. Identifying the target audience of this campaign is a crucial first step in raising awareness of ATRs. Literature shows that warm-up periods before any physical activity and regular exercise are strategies that can be implemented to avoid ATR [25]. If a significant proportion of ATRs occurred when playing football, then promoting a warm-up habit before playing on a football pitch could decrease ATR incidence.

## Conclusions

Within this study's sample, ATRs were more common in males (76%), were predominantly sports-related (67%), with football (39%) being the most common activity of all ATRs in this study. The most prevalent mechanism of injury was acceleration from a stationary position (30%). ATRs were found to have a higher incidence during summer, but there was no statistical seasonal variation of ATRs in this sample. 24% of participants reported preceding ankle pain, although only 1% had a previous ipsilateral ATR. All other risk factors and comorbidities were found to have a greater incidence in NSRATRs. Most participants were managed conservatively (90%), and only 5% experienced re-ruptures.

This study highlights a need to increase ATR epidemiology data nationally, which could be used to guide clinical judgement and develop targeted injury prevention strategies.

## Appendices

Table 4 shows the telephone consultation proforma used to clarify and confirm data that could not be ascertained from patient records.

**Table 4:** Telephone Consultation Proforma The data collected from telephone consultations

1. Patient Name:	
2. Date of Call:	
3. Call Attempt Number:	
4. Date of injury:	
5. Date of presentation to Accident and Emergency Department (A&E):	
6. Ankle Affected:	
7. Activity of Injury:	
7a. If True, Route of Administration	
8. Mechanism of Injury:	
9. Weight bearing:	
10. Preceding Ankle Pain:	
10a. If True, Duration in days	
10b. If True, Activity of Onset of Pain	
11. Previous Achilles Tendon Rupture	
11a. If True, Date of Previous Rupture	
11b. If True, Ankle Affected	
11c. If True, Activity of Injury	
11d. If True, Mechanism of Injury	
12. Smoking Status	
13. Statin Use at Time of Injury	
14. Ciprofloxacin Use near Date of Injury	
15. Steroid Use at Time of Injury	
16. Re-rupture	
16a. If True, Date of Re-rupture	
16b. If True, Activity of Injury	
16c. If True, Mechanism of Injury	
